# Trend of Soil-Transmitted Helminths in Ethiopian Children: A Systematic Review and Meta-Analysis (2000-2018)

**DOI:** 10.1155/2021/5638836

**Published:** 2021-10-22

**Authors:** Getaneh Alemu, Feleke Mekonnen, Mezgebu Nega, Chalachew Muluneh

**Affiliations:** ^1^Department of Medical Laboratory Science, Bahir Dar University, Bahir Dar, Ethiopia; ^2^Amhara National Regional State Health Bureau, Bahir Dar, Ethiopia

## Abstract

**Background:**

Ethiopia is one of the tropical countries with a heavy burden of soil-transmitted helminths. As a result, the nation has been implementing mass drug administration, water, sanitation, and hygiene and health extension programs to control those parasites. Hence, updated data about the prevalence and trend of parasites over time has a pivotal role to assess the success of existing control programs.

**Methods:**

Studies conducted between 2000 and 2018 were searched from PubMed, Google Scholar, and local journals for systematic reviews and meta-analysis following the PRISMA guideline and checklists. Eligible studies were selected based on preset inclusion and exclusion criteria. The quality of the included studies was assessed using the Newcastle-Ottawa Scale in meta-analysis. Heterogeneity between studies was assessed using the Cochran *Q* test and *I*^2^ test statistics based on the random effect model. Comprehensive meta-analysis (CMA 2.0) was used to calculate the pooled prevalence, and metaregression was run to assess the trend of parasite prevalence over time.

**Results:**

Thirty-eight studies recruiting 16,266 participants were included in the review. The pooled prevalence of intestinal parasites was 52.0% (95% CI: 44.4-59.5). Amhara region was with the highest prevalence (60.3%; 95% CI: 50.1-69.6). Among soil-transmitted helminths, *Ascaris lumbricoides* (11.2%; 95% CI: 8.4-14.8) was with the highest pooled prevalence followed by hookworms (10.4%; 95% CI: 7.9-13.7) and *Trichuris trichiura* (3.6%; 95% CI: 2.4-5.4). Metaregression analysis revealed that all soil-transmitted helminths did not show a significantly decreasing trend over time (*p* > 0.05).

**Conclusion:**

Despite various control efforts having been made, soil-transmitted helminths are of high distribution, and their prevalence is not significantly decreasing in Ethiopia. Hence, other control approaches like community-led sanitation should be integrated with mass drug administration to achieve the national goal of soil-transmitted helminth elimination by 2025.

## 1. Background

Intestinal parasitic infections (IPIs) are among the major public health problems worldwide. Globally, 3.5 billion people are infected, among which 450 million manifest illnesses because of the infection [1]. Poor sanitary conditions and conducive climate for the survival of parasites outside the host make the majority of intestinal parasites (IPs) abundant in the tropics [2]. Globally, *Ascaris lumbricoides* (*A. lumbricoides*), *Trichuris trichiura* (*T. trichiura*), and hookworms infect 819, 465, and 439 million people, respectively. They are grouped under soil-transmitted helminths (STHs) because their infective stages embryonate or develop in the soil [3].

In Ethiopia, 81 million people are at risk of STH infection. School-age children (SAC) account for nearly one-third (25.3 million) of the risk population [4]. Transmission of STHs is associated with personal hygiene and sanitation practices as well as access to safe water. However, the Ethiopian Demography and Health Survey report shows that 40% and 33% of households had no hand washing facilities and toilets at their home. Access to safe water for drinking was also poor in the rural community with only 57% coverage [5]. These conditions contribute to ease of transmission for IPs in general and STHs in particular [6].

Even if all population groups can be infected by STHs, SAC are the most commonly affected groups. This is because of their immature immune system and frequent exposure to infective sources (soil and water) as well as poor hygiene and sanitary practices [7, 8]. Moreover, children are more vulnerable to serious complications such as prolonged diarrhea, malnutrition, anemia, bowel obstruction, and learning disabilities compared to adults [9]. Hence, the Federal Ministry of Health of Ethiopia has been taking strong measures targeted for the control of IPs among SAC. Implementation of the health extension program since 2003/4, which focuses on the preventive health packages, has brought tangible improvements in the health of the rural community. Creating awareness about latrine construction and utilization as well as keeping personal and environmental hygiene among the community has been one of the priority concerns of the program [10]. Implementation of biannual mass drug administration (MDA) with albendazole or mebendazole for the control of STH since 2015, primarily targeting SAC, has been the other important commitment of the ministry [11].

All these efforts are expected to reduce the burden and health impacts of STHs from time to time. Several survey studies have been conducted among SAC at different geographical settings of the country. However, there is no adequate data reporting the pooled prevalence and trend in magnitude of STHs over time. Hence, we conducted a systematic review and meta-analysis aimed at assessing the pooled prevalence and trend of STH infections among SAC in Ethiopia. The review provides evidence about the impact of ongoing control and prevention programs and to plan for the future. In this review, we did not include *Strongyloides stercoralis* because we need to focus on the main STHs which have been targeted for MDA-based intervention so that we can analyze the impact of MDA on their prevalence. Moreover, there have been controversies whether *Strongyloides stercoralis* fulfills the definition of STHs.

## 2. Methods

### 2.1. Setting

We included studies conducted all over Ethiopia. Ethiopia is located in the horn of Africa at geographical coordinates of 8° N and 38° E [12]. The country is composed of nine regional states and two city administration councils. Altitude of the country ranges from high peaks of 4,620 meters above sea level to a low depression of 148 meters below sea level. More than half of the country lies above 1,500 meters [4, 12]. The estimated population of the country for 2018 was 107.53 million where SAC of age 5-14 years account for 31.2% [4, 13].

### 2.2. Eligibility Criteria

School- or community-based studies conducted in Ethiopia between January 2000 and December 2018 targeting SAC (4-25 years old) and published in the English language were included in the present review. Studies reporting both intestinal protozoa and helminths or intestinal helminths alone were included since our primary targets were STHs.

### 2.3. Information Sources and Search of Literature

Potential articles were searched in PubMed, Google Scholar, and local journals following the PRISMA guideline and checklists [14]. Search in PubMed was done using the MeSH terms “helminth OR intestinal helminth OR helminthiasis OR intestinal parasite OR parasite OR parasitosis OR intestinal parasitosis OR parasitic infection AND Ethiopia.” A manual search was also conducted on all relevant references listed within the articles identified after an initial electronic search. The search was independently done by two reviewers to minimize bias and missing of studies.

### 2.4. Study Selection

**Figure 1:**
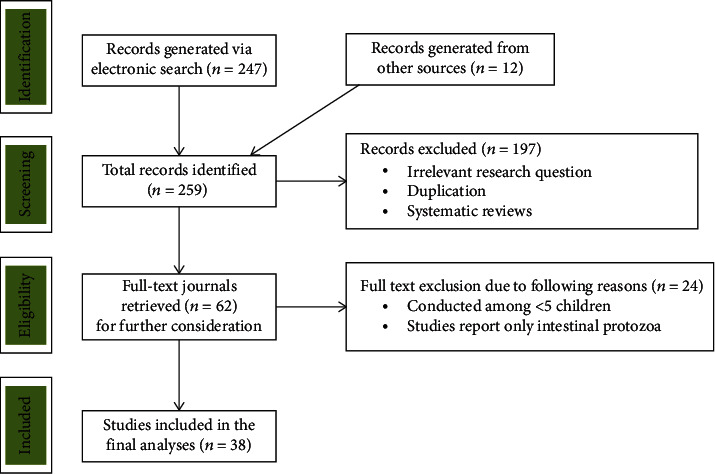
Flow chart showing selection process of eligible studies.

The study selection process is shown in Figure 1. Studies conducted in health facilities were excluded because we expect inflated data as only symptomatic children with parasitic illness participate in healthcare facility-based studies. Because our interest in this review was the SAC, the most susceptible group for IPI, studies targeting preschool children and adults alone were not considered. Surveys conducted before January 2000 and those reporting only intestinal protozoa were also excluded from the review. Published surveys reporting at least the age group of study participants, location of data collection, and data collection period were included in this review (Figure 1). The search was conducted by two authors separately, and there were differences in the inclusion of the three studies. After discussion, both authors agreed to include two and excluded one study.

### 2.5. Data Extraction

Two authors extracted data independently using a standard data collection form constructed in Excel. Information was collected about the total number of children that participated, total number of children with laboratory confirmed intestinal parasitosis, age group, year of study, location of sampling (study region), laboratory methods used for investigation of fecal specimens, groups of parasites detected (helminth only/helminth and protozoa), and total number of children infected with the big three STHs (*A. lumbricoides*, hookworms, and *T. trichiura*).

### 2.6. Statistical Analysis

Estimation of the pooled prevalence of IPIs and summary prevalence of each STH was calculated using CMA 2.0 software. Subgroup analysis was done by region of study and study period. The random effect model was used in the analysis because the study populations across selected studies vary, at least in terms of age group and level of risk of IPI. Heterogeneity between studies was checked with a forest plot, Cochran's *Q* test, and *I*^2^ test. Significant heterogeneity was declared at *I*^2^ > 50% and *Q* test (*p* < 0.10). Quality of study was checked with the Newcastle-Ottawa Scale adapted for cross-sectional study by two reviewers independently, and disagreements were resolved by discussion.

### 2.7. Risk of Bias across Studies

Presence of publication bias was assessed by drawing funnel plots. Logit event rates of the studies were plotted against standard error so that asymmetry in the distribution of studies can easily be observed. For further assessment of publication bias, Begg's adjusted rank correlation and Egger's regression asymmetry test were also used. Significant publication bias was considered if *p* < 0.05 in those tests. Leave-one-out analysis was done to assess the outlier study results responsible for the skewed pooled prevalence of intestinal parasites.

## 3. Results

### 3.1. Characteristics of Studies

Among the initially identified 259 studies, 38 cross-sectional studies conducted from the years 2000 to 2018 were included in the analysis (Figure 1). A total of 16,266 participants with 4 to 25 years of age range were recruited in the study. The smallest and largest sample sizes among the included studies were 279 and 855, respectively [15, 16]. The lowest and the highest IP prevalence among the studies were 7.1% and 85.4%, respectively [17, 18]. Nearly half (16 out of 38) of the studies were conducted in the Amhara region. Twenty-five studies reported only intestinal helminths [16–40], while the remaining 13 studies reported both intestinal helminths and protozoa [15, 41–52]. All studies were school-based [15–45, 47–52] except 1 which was conducted at the community level [46]. Eleven and nine studies screened stools by formol ether concentration and Kato-Katz techniques, respectively, while seven studies performed both direct wet mount and formol ether concentrations (Table 1).

### 3.2. Prevalence of Intestinal Parasites

**Figure 2:**
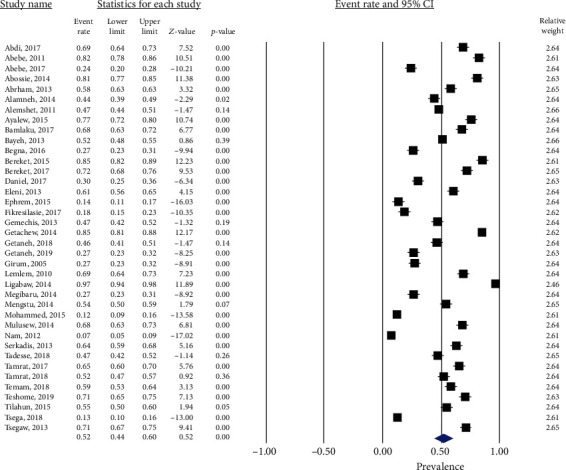
Forest plot showing pooled prevalence of intestinal parasites.

**Figure 3:**
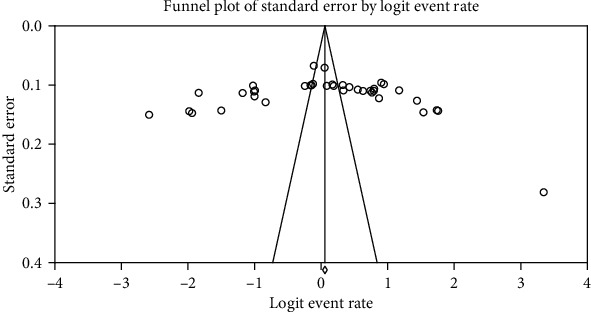
Publication bias assessment funnel plot; Egger's regression test (*p* = 0.481) and Begg's rank correlation (*p* = 0.470).

Among a total of 16,266 children participated; 8,200 were infected with at least one IP, yielding a pooled prevalence of 52.0% (95% CI: 44.4-59.5.0; *I*^2^ = 98.7, *p* < 0.001) (Figure 2). Analysis with stepwise removal of each study revealed a pooled prevalence between 50% and 53.8%. Pooled prevalence of IPs among studies reporting both intestinal helminths and protozoa was 53.0% (95% CI: 41.0-64.0; *I*^2^ = 98.4, *p* < 0.001). Similarly, pooled prevalence of IPs among studies reporting only intestinal helminths was 52.0% (95% CI: 42.0-62.0; *I*^2^ = 98.8, *p* < 0.001). Distribution of studies in the funnel plot, Egger's regression (*p* = 0.481), and Begg's correlation (*p* = 0.470) showed that there was no significant publication bias among the included studies (Figure 3).

### 3.3. Subgroup Prevalence of Intestinal Parasites

**Figure 4:**
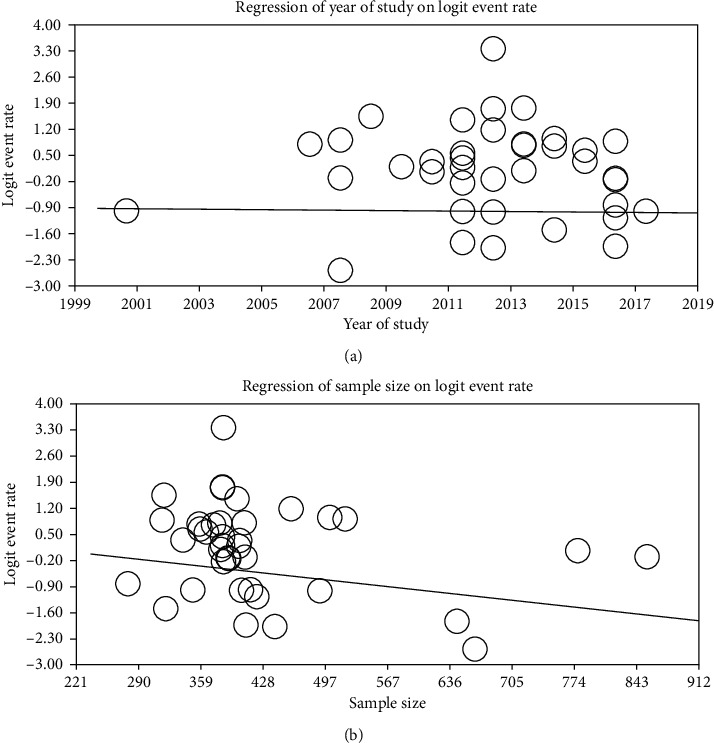
Metaregression of prevalence of IPs by (a) year of study (*B* = −0.00487, *p* = 0.458) and (b) sample size (*B* = −0.00252, *p* = 0.057).

Prevalence of IPs was analyzed by region of study and study period. Region-wise analysis has revealed a pooled prevalence of 60.3% (95% CI: 50.1-69.6; *I*^2^ = 98.3, *p* < 0.001), 39.7% (95% CI: 27.4-53.5; *I*^2^ = 98.5, *p* < 0.001), 62.1% (95% CI: 44.6-76.9; *I*^2^ = 98.8, *p* < 0.001), and 34.0% (95% CI: 11.1-67.9; *I*^2^ = 99.3, *p* < 0.001) in Amhara, Oromia, Southern Nations Nationalities and People's Region (SNNPR), and Tigray, respectively. Subgroup analysis by study period has shown that the pooled prevalence of IPs between 2000 and 2013 was 52.2% (95% CI: 41.4-62.7; *I*^2^ = 98.7, *p* < 0.001) while it was 51.9% (95% CI: 41.2-62.4; *I*^2^ = 98.4, *p* < 0.001) for studies conducted from 2014 to 2020. Metaregression analysis show that there was no significant trend in decreasing the prevalence of IPs by study period and sample size (Figure 4).

### 3.4. Prevalence and Trend of Soil-Transmitted Helminths

**Figure 5:**
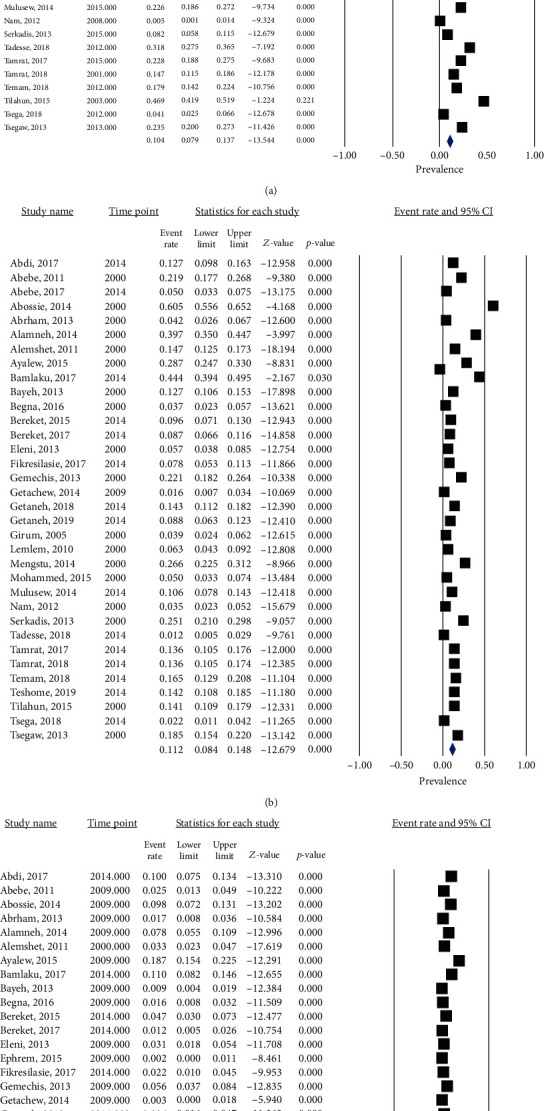
Forest plot showing pooled prevalence of (a) hookworms, (b) *A. lumbricoides*, and (c) *T. trichiura*.

**Figure 6:**
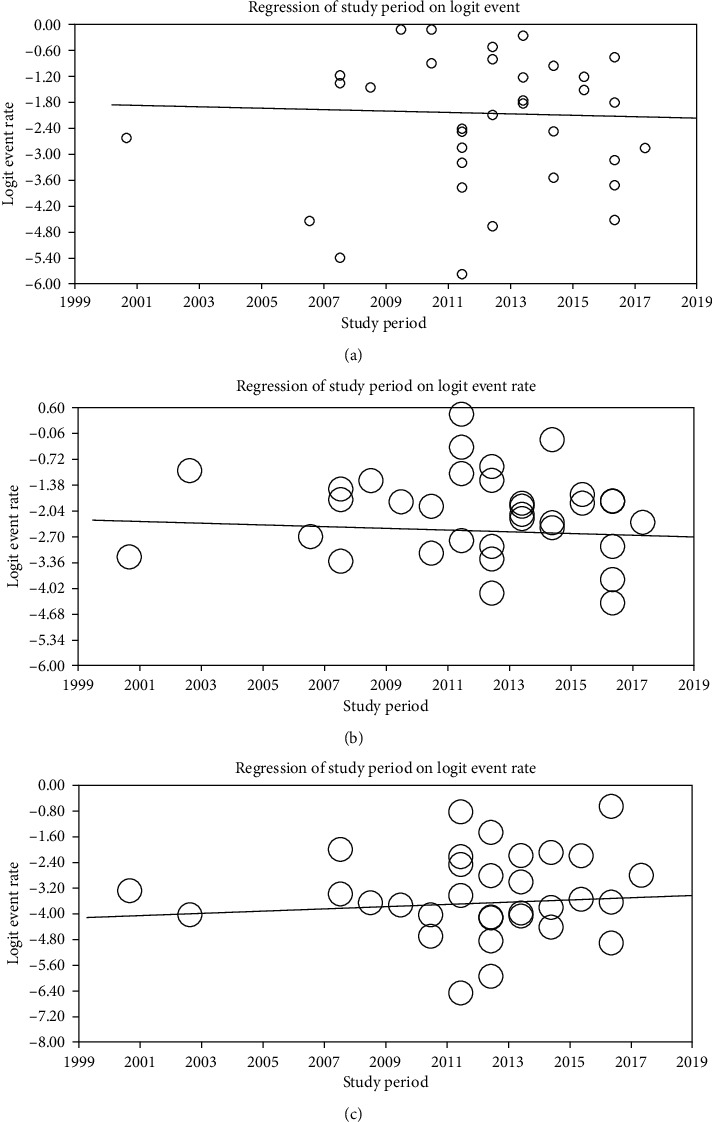
Metaregression of prevalence of (a) hookworms (*B* = −0.01141, *p* = 0.138), (b) *A. lumbricoides* (*B* = −0.02173, *p* = 0.610), and (c) *T. trichiura* (*B* = 0.0326, *p* = 0.565) by study period.

Thirty-four studies have reported hookworms giving a pooled prevalence of 10.4% (95% CI: 7.9-13.7, *I*^2^ = 97.45, *p* < 0.001). The prevalence of hookworms was 11.7% (95% CI: 8.0-16.8) between 2000 and 2013 and 8.7% (95% CI: 6.5-13.4) between the years 2014 and 2020. Analysis of hookworm prevalence has shown a slightly decreasing trend, but it was not statistically significant (*p* = 0.138). The pooled prevalence of *A. lumbricoides* and *T. trichiura* was 11.2% (95% CI: 8.4-14.8, *I*^2^ = 97.5, *p* < 0.001) and 3.6% (95% CI: 2.4-5.4; *I*^2^ = 96.3, *p* < 0.001), respectively (Table 2; Figures 5 and 6).

## 4. Discussion

The present systematic review and meta-analysis was designed to generate comprehensive data about the national prevalence of IPs in general and STHs in particular. Accordingly, studies assessing the prevalence of IPIs, conducted in different regions of Ethiopia, were gathered and analyzed to estimate the national pooled prevalence. The findings provide useful epidemiological data to aid in the control of STHs. The review generated information about the distribution of the big three STHs: *A. lumbricoides*, hookworm, and *T. trichiura* in Ethiopia which, in turn, helps to evaluate the success of existing control programs and to plan for the future. It also helps to implement targeted control activities. The overall pooled prevalence of IPs in the present review (52.0%) was similar to the 47.6% prevalence in Afghanistan [53] but higher than the prevalence in Iran (38%) [54], Syria (42.5%) [55], Turkey (31.8–37.2%) [1], and Egypt (27%) [56]. Variations in the distribution of IPs among different geographical settings as well as the type and level of control program implementation across countries might be responsible for these differences.

The pooled prevalence of hookworms in the present review (10.4%) was in line with review results from South America (11.9%) [57]. It was lower than the Ethiopian national estimate of 16% before 10 years [58] and a region-wide survey in the Amhara region (20.6%) [59] pronounced the impact of MDA and WASH activities since then. Subgroup analysis of the current review also shows the highest prevalence of IPs in the Amhara region. The prevalence was also lower than the pooled prevalence from Nigeria (23.0%) [54] and Rwanda (31.6%) [60]. The cumulative number of children included in the review from Nigeria was higher compared to the present review (34,518 vs. 16,266) [54]. Moreover, we have included more recent surveys that the ongoing MDA and WASH programs also impact the prevalence of hookworms unlike in reviews from Rwanda which included studies starting from the year 1940 [60].

Implementation of intervention programs mainly improved healthcare coverage and biannual MDA, and WASH has been thought to bring a decreasing trend of both morbidity and prevalence of STH infections over time. As a result, hookworms show a decreased prevalence between the years 2014 and 2020 (8.7%) compared to that of the years 2000-2013 (11.7%). However, the trend in decrement was not significant (*p* = 0.138). This is to the review results from Nepal where hookworm infections significantly decrease between the years 1990 and 2015 [61]. Interventions for STH transmission in Ethiopia primarily target SAC. However, in rural areas where fields are fertilized with night soil, STH species like hookworms may heavily infect adults who, in turn, serve as sources of infection for SAC [59]. Open defecation is common in rural communities of Ethiopia that reinfection of treated children also contributed to the nondecreasing trend of the parasite.

The pooled prevalence was higher than findings from nationwide surveys in Sri Lanka (1.2%) [62] and Cameroon (1.55%) [63]. In both countries, the authors used a single Kato-Katz smear, and there is also variation in geographical distribution as well as adoption and level of implementation of control programs across countries.

The pooled prevalence of *A. lumbricoides* in the present review (11.2%) was in line with findings from Cameroon (11.48%) [63]. However, it was lower than results from Nigeria (44.6%) [64], Rwanda (38.6%) [60], South America (15.6%) [57], the Amhara region of Ethiopia (16.8%) [59], and a previous estimate in Ethiopia which was 37% [58]. Local studies about mebendazole and albendazole efficacy show that both drugs have more than 95% efficacy against *A. lumbricoides* and that the ongoing MDA has substantially decreased the burden of the parasite in Ethiopia [65]. However, the trend of *A. lumbricoides* was not uniformly decreasing between the years 2000 and 2020 (*p* = 0.610) with the possible reason of poor WASH implementation in the country [5]. The pooled prevalence of *A. lumbricoides* in the present review was higher than the review result of 0.75% from Iran [54] and 2.8% from Sri Lanka [62].

The pooled prevalence of *T. trichiura* in the present review (3.6%) was in line with reviews from the Amhara region (3.8%) [59] and Sri Lanka which was 4% [62]. On the other hand, it was lower than review results from South America (12.5%) [57], Nigeria (31.9%) [64], Cameroon (18.22%) [63], Rwanda (27%) [60], and previous estimates in Ethiopia (30%) [58]. The pooled prevalence of *T. trichiura* in the present review was higher than the review result of 0.12% from Iran [54]. Metaregression analysis by year of study showed that *T. trichiura* has almost constant prevalence between the years 2000 and 2020 in Ethiopia. Both albendazole and mebendazole, drugs used for MDA, have poor efficacy against *T. trichiura* contributing to the nondecreasing trend of parasite prevalence [65].

### 4.1. Strength and Limitations

The strength of the present review is that it included a large number of studies and has identified the pooled prevalence of the big three STHs which are targeted for control in Ethiopia. As a limitation, the studies included in the present review were conducted in only five regions and we could not get studies from other regions. The primary targets of this review were SAC; however, many studies have included adolescents. Considering a very small proportion of participants are above the age limit of SAC and with the concern not to miss potential findings, we included studies which recruited participants of age 4-25 years old. There was also variation in laboratory techniques used across the reviewed studies. The pooled total IP prevalence in the present study might not show the exact figure in the country because studies not reporting STH were excluded as STHs were the focus of the present review [66].

## 5. Conclusion

The prevalence of IPs in Ethiopia is unacceptably high infecting more than half of the population. This is against the national goal. The federal Ministry of Health has set a goal to eliminate the three STHs (ascariasis, hookworm infection, and trichuriasis) as public health problems by 2025. Despite various control efforts having been made, STHs are with high distribution and their prevalence is not significantly decreasing in Ethiopia. Hence, the integration of the existing control activities (MDA and WASH) accompanied with continuous efforts to create awareness and engagement among the community should be promoted to achieve the national goal of STH elimination by 2025. Alternative treatment options like combination therapy of albendazole/mebendazole with ivermectin should be considered for trichuriasis.

## Figures and Tables

**Table 1:** Characteristics of included studies (*n* = 38).

Study name	Study region	Study period	Sample size	Total positive	IP prevalence	Lab method	IP detected	Age group
Merem, 2017 [19]	Amhara	2013-14	408	282	69.1	FEC	Helminth	6-20
Abebe, 2011 [20]	Amhara	2009	319	263	82.4	KATO	Helminth	5-19
Abebe, 2017 [41]	AA	2017	422	100	23.7	DWM, FEC	Both	4-18
Ashenafi, 2014 [42]	SNNPR	2012	400	324	81.0	DWM, FEC	Both	5-15
Abraham, 2013 [21]	Amhara	2011	403	235	58.3	KATO	Helminth	5-15
Alamneh, 2014 [22]	Amhara	2011-12	385	170	44.2	FEC	Helminth	6-18
Alemeshet, 2011 [16]	Oromia	2008	855	406	47.5	FEC	Helminth	7-16
Ayalew, 2015 [43]	SNNPR	2013	460	352	76.5	KATO	Both	5-17
Bamlaku, 2017 [23]	SNNPR	2015	374	254	67.9	FEC	Helminth	5-15
Bayeh, 2013 [24]	Amhara	2011	778	401	51.5	KATO	Helminth	7-14
Begna, 2016 [44]	Oromia	2013	492	131	26.6	DWM, FEC	Both	6-19
Bereket, 2015 [18]	SNNPR	2014	384	328	85.4	KATO, SAF	Helminth	5-19
Bereket, 2017 [25]	SNNPR	2015	503	363	72.2	KATO, SAF	Helminth	5-19
Daniel, 2017 [15]	Amhara	2017	279	85	30.5	FEC	Both	6-16
Eleni, 2013 [45]	Tigray	2011-12	384	233	60.7	DWM	Both	6-18
Ephrem, 2015 [26]	Oromia	2012	644	89	13.8	McMaster	Helminth	5-25
Fikreslasie, 2017 [27]	Oromia	2014-15	321	59	18.4	FEC	Helminth	6-15
Gemechis, 2013 [28]	Oromia	2013	390	182	46.7	KATO, FEC	Helminth	6-17
Getachew, 2014 [29]	Amhara	2013	384	327	85.2	KATO, SAF	Helminth	5-19
Getaneh, 2018 [46]	SNNPR	2017	391	181	46.3	FEC	Both	5-14
Getaneh, 2019 [47]	SNNPR	2018	351	95	27.1	DWM, FEC	Both	5-14
Girum, 2005 [30]	Oromia	2001	415	113	27.2	FEC	Helminth	5-24
Lemlem, 2010 [48]	Tigray	2007	381	263	69.0	KATO	Both	5-19
Ligabaw, 2014 [31]	Amhara	2013	385	372	84.9	FEC, KATO	Helminth	6-15
Megbaru, 2014 [32]	SNNPR	2012	405	109	26.9	KATO	Helminth	5-18
Mengstu, 2014 [33]	Amhara	2011-12	402	219	54.5	DWM, FEC	Helminth	6-19
Mohammed, 2015 [34]	Tigray	2013	442	54	12.8	KATO	Helminth	5-20
Mulusew, 2014 [49]	Amhara	2014	358	245	68.4	DWM, FEC	Both	7-21
Nam Linh, 2012 [17]	Amhara	2008	664	47	7.1	FEC	Helminth	6-19
Serkadis, 2013 [35]	Oromia	2011-12	366	233	63.7	McMaster	Helminth	5-15
Tadesse, 2018 [50]	Amhara	2017	409	193	47.2	Richie's	Both	7-14
Tamirat, 2017 [36]	Amhara	2015-16	359	235	65.5	FEC	Helminth	7-14
Tamirat, 2018 [51]	Amhara	2014	382	200	52.4	FEC	Both	7-13
Temam, 2018 [37]	Oromia	2016	340	199	58.5	DWM, KATO	Helminth	6-19
Teshome, 2019 [38]	Oromia	2017	317	224	70.7	KATO, FEC	Helminth	5-15
Tilahun, 2015 [39]	Amhara	2010	384	211	54.9	KATO	Helminth	5-14
Tsega, 2018 [40]	Tigray	2017	410	52	12.7	KATO	Helminth	6-19
Tsegaw, 2013 [52]	Amhara	2008	520	371	71.3	DWM, FEC	Both	6-15

AA: Addis Ababa; DWM: direct wet mount; FEC: formol-ether concentration; KATO: Kato-Katz; SNNPR: Southern Nations Nationalities and People's Region.

**Table 2:** Metaregression analysis of prevalence of STHs by study period.

Parasite species	No. of studies	Pooled prevalence % (95% CI)			*β*	*p* value
		2000-2013	2014-2018	Overall		
Hookworms	34	11.7 (8.0-16.8)	8.7 (6.5-13.4)	10.4 (7.9-13.7)	-0.01141	0.138
*A. lumbricoides*	34	12.2 (8.2-17.9)	10.0 (6.7-14.6)	11.2 (8.4-14.8)	-0.02173	0.610
*T. trichiura*	32	3.2 (1.5-5.5)	4.2 (2.2-8.0)	3.6 (2.4-5.4)	0.0326	0.565

## Data Availability

The original data for this study is available from the corresponding author.

## Abbreviations

IPIs: Intestinal parasitic infections
IPs: Intestinal parasites
MDA: Mass drug administration
SAC: School-age children
STHs: Soil-transmitted helminths
WASH: Water hygiene and sanitation.

## Supplementary Materials

Supplementary Materials PRISMA checklist.
